# CCL20 induced by visfatin in macrophages via the NF-κB and MKK3/6-p38 signaling pathways contributes to hepatic stellate cell activation

**DOI:** 10.1007/s11033-020-05510-7

**Published:** 2020-05-16

**Authors:** Yu Jung Heo, Sung-E Choi, Nami Lee, Ja Young Jeon, Seung Jin Han, Dae Jung Kim, Yup Kang, Kwan Woo Lee, Hae Jin Kim

**Affiliations:** 1 Department of Biomedical Sciences, Graduate School of Ajou University, Suwon, Republic of Korea; 2 Department of Physiology, Ajou University School of Medicine, Suwon, Republic of Korea; 3 Department of Endocrinology and Metabolism, Ajou University School of Medicine, 206, World cup-ro, Yeongtong-gu, Suwon, 16499 Republic of Korea

**Keywords:** Visfatin, CCL20, Fibrosis, MKK3/6-p38

## Abstract

Chemokines interact with hepatic resident cells during inflammation and fibrosis. CC chemokine ligand (CCL) 20 has been reported to be important in inflammation and fibrosis in the liver. We hypothesized that visfatin, an adipocytokine, could play a role in hepatic fibrosis via CCL20. We investigated the effect of visfatin on CCL20 in THP-1 human promonocytic cells and examined the molecular mechanisms involved. Following treatment of THP-1 cells with visfatin, CCL20 expression and secretion were assessed. We assessed the intracellular signaling molecules IKK/NF-κB, JAK2/STAT3, MAPKs, and MKK3/6 by western blotting. We treated THP-1 cells with visfatin and signaling inhibitors, and examined CCL20 mRNA and protein levels. To investigate the effect of visfatin-induced CCL20 expression in hepatic stellate cells (HSCs), LX-2 cells were co-cultured with the culture supernatant of THP-1 cells with or without anti-CCL20 neutralizing antibodies, and fibrosis markers were examined by RT-PCR and immunoblotting. In THP-1 cells, visfatin increased the CCL20 mRNA and protein levels. visfatin increased the activities of the NF-κB, p38, and MLK3/6 signaling pathways but not those of the JAK2/STAT3 and ERK pathways. Visfatin treatment together with an NF-κB, p38, or MLK3 inhibitor reduced the mRNA and protein levels of CCL20. The visfatin-induced CCL20 increased the expression of fibrosis markers and CCR6 in HSCs. Following neutralization of CCL20, the levels of fibrosis markers and CCR6 were decreased. Visfatin increases the expression of CCL20 via the NF-κB and MKK3/6-p38 signaling pathways in macrophages, and visfatin-induced CCL20 expression promotes the fibrosis markers in HSCs.

## Introduction

Non-alcoholic fatty liver disease (NAFLD) is a broad range of conditions ranging from fatty liver to non-alcoholic steatohepatitis (NASH), fibrosis, and cirrhosis [1]. Progression of NAFLD is a complex process involving the interaction of a number of cell types, including hepatocytes, Kupffer cells, hepatic stellate cells (HSCs), and infiltrating immune cells [2]. The signals that regulate this complex multicellular process are unclear [3], but likely involve production of a number of proinflammatory mediators, such as cytokines and chemokines [4].

Chemokines are a family of small cytokines that have the properties of both chemotactic mediators and cytokines [5]. Chemokines mediate the infiltration of immune cells into the injured liver but can also directly interact with hepatic resident cells during inflammation and fibrosis [6]. CC chemokine ligand 20 (CCL20) was originally identified as a liver- and activation-related chemokine in the liver, and is also known as a macrophage inflammatory protein (MIP-3α) [7]. CCL20 has been described as the only chemokine that interacts with and activates CC chemokine receptor 6 (CCR6) [8]. CCL20 has been reported to be important in inflammation and fibrosis in the liver [9, 10]. However, the mechanism of CCL20 in hepatic inflammation and liver fibrosis is unclear.

Li et al. [11] reported the effect of chemokine CCL20 mRNA and protein levels in human monocyte-derived macrophages of plasmin, which is dependent on activation of the p38 mitogen-activated protein kinase (MAPK) and nuclear factor (NF)-κB signaling pathways. Brand et al. [12] reported that CCL20 activated the Akt, extracellular signal-regulated kinase (ERK) 1/2, and stress-activated protein kinase/c-Jun N-terminal kinase (JNK) MAPK pathways. Several groups of MAPK signal transduction pathways have been identified in mammals, including the ERK, JNK, and p38 MAPK pathways. The phosphorylation is mediated by seven mitogen-activated protein kinases kinases (MAPKKs, MKKs) specific for individual MAPK isoforms. Thus, ERK1/2 are activated by MAP kinase/ERK kinases MEK1 and MEK2, ERK5 is activated by MEK5, JNK is activated by MKK4 and MKK7, and p38 MAPK is activated by MKK3 and MKK6 [13, 14].

The adipocytokine visfatin, also known as pre-B cell colony-enhancing factor (PBEF), plays important roles in immunity and inflammation [15]. Visfatin also functions as an immunomodulatory cytokine, triggering the production of cytokines such as interleukin (IL)-1, IL-6, tumor necrosis factor (TNF)-α, and IL-8, which induces chemotaxis of activated mononuclear cells [16–18]. Moreover, visfatin might affect organ fibrosis [19–21]. However, the signaling pathways involved in the effect of visfatin in macrophages are unclear, as is its role in hepatic inflammation and fibrosis.

We hypothesized that visfatin could play a role in hepatic fibrosis via CCL20. We evaluated the signaling pathways involved in visfatin-induced CCL20 production in macrophages.

## Materials and methods

### Materials

Recombinant human visfatin was purchased from PeproTech (Rocky Hill, NJ, USA). The mixed lineage kinase (MLK) inhibitor, URMC-099, was purchased from Cayman Chemical (Ann Arbor, MI, USA). The NF-κB inhibitor, Bay 7082, was purchased from Calbiochem (San Diego, CA, USA). The p38 MAPK inhibitor, SB203580, and the JNK inhibitor, SP600125, were purchased from Sigma (St. Louis, MO, USA). The anti-CCL20 neutralizing antibody was purchased from R&D Systems (Minneapolis, MN, USA). Antibodies against IκB kinase (IKK), phospho-IKKα/β, NF-κB, phospho-NF-κB, Janus kinase 2 (JAK2), phospho-JAK2, signal transducer and activator of transcription 3 (STAT3), phospho-STAT3, JNK, phospho-JNK, p38, phospho-p38, MKK3, MKK6, and phospho-MKK3/6 were purchased from Cell Signaling Technology (Danvers, MA, USA). Anti-actin, phospho-ERK, and anti-ERK connective tissue growth factor (CTGF) antibodies were purchased from Santa Cruz Biotechnology (Santa Cruz, CA, USA). Anti-collagen III and anti-fibronectin antibodies were purchased from Abcam (Cambridge, MA, USA). Culture media, culture supplements, and fetal bovine serum (FBS) were obtained from Gibco-BRL (Grand Island, NY, USA).

### Cell culture

THP-1 cells, a promonocytic cell line, were obtained from the American Type Culture Collection (Manassas, VA, USA) and cultured in Roswell Park Memorial Institute (RPMI) medium 1640 (Life Technologies, Grand Island, NY, USA). THP-1 monocytes were differentiated into macrophages by 24 h incubation with 100 nM phorbol 12-myristate 13-acetate (PMA; Sigma) followed by 24 h incubation in RPMI medium. Following differentiation into macrophages, cells were incubated with 10 pg/mL lipopolysaccharide (LPS) from Escherichia coli O111:B4 (Sigma) and stimulants. LX-2 cells, immortalized human HSCs, were kindly provided by Professor Sang Geon Kim (College of Pharmacy, Seoul National University, Seoul, South Korea). LX-2 cells were grown in Dulbecco’s modified Eagle’s medium (DMEM; Hyclone, Waltham, MA, USA) supplemented with 10% fetal bovine serum (FBS; Equitech-bio, Kerrville, TX, USA) and 1% antibiotics (Hyclone) in an atmosphere of 5% CO2 at 37 °C. The medium was supplemented with 10% (v/v) FBS and antibiotics (10 μg/mL streptomycin and 100 IU/mL penicillin) at 37 °C in a humidified atmosphere of 95% air and 5% CO2 (both v/v). LX-2 cells were incubated with transforming growth factor (TGF)-β for 24 h prior to treatment with the culture supernatant of THP-1 cells. Before treatment with 0.1% bovine serum albumin (BSA)/phosphate-buffered saline (PBS) or visfatin (dissolved in 0.1% BSA/PBS), the medium were replaced with serum-free medium.

### Immunoblotting

Cells were suspended in RIPA buffer by adding a protease inhibitor cocktail (Roche Applied Science, Mannheim, Germany). The protein concentrations in lysates were determined using a protein assay kit (Bio-Rad, Hercules, CA, USA). Equal volumes of 2× sodium dodecyl sulfate (SDS) sample buffer (125 mM Tris–HCl [pH 6.8], 4% [w/v] SDS, 4% [v/v] 2-mercaptoethanol, and 20% [v/v] glycerol]) were added to the lysates, and equivalent amounts of protein (30 µg) were loaded, electrophoresed, and electrophoretically transferred to polyvinylidene difluoride membranes (Millipore, Billerica, MA, USA) [19]. After blocking with 5% (w/v) skim milk or BSA, the target antigens were reacted with primary antibodies, followed by the addition of secondary antibodies (horseradish peroxidase-conjugated anti-goat IgG or anti-rabbit IgG). Immunoreactive bands were visualized using an enhanced chemiluminescence kit from Amersham Pharmacia Biotech (Piscataway, NJ, USA).

### RNA isolation and quantitative real-time polymerase chain reaction (PCR)

Total cellular RNA was isolated using RNAiso Plus reagent (TaKaRa Bio, Otsu, Japan) according to the manufacturer’s instructions. Briefly, cDNA was prepared from THP-1 and LX-2 cells using avian myeloblastosis virus reverse transcriptase and random 9-mer primers. The cDNA was amplified by qPCR using primer sets specific for human CCL20: TTC ACC CAA GTC TGT TTT GGA (forward [F]) and GAA GGC TGT GAC ATC AAT GCT (reverse [R]); human CCR6: GCC TGA ACC CTG TGC TCT AC (F) and CAC AGG AGA AGC CTG AGG AC (R); human collagen I: AAT CCA TCG GTC ATG CTC TC (F) and GGC CCA GAA GAA CTG GTA CA (R); human MMP2: GGT GCT GGC TGA GTA GAT CC (F) and AGC TCC CGG AAA AGA TTG AT (R); and human TIMP1: TGC AGT TTT CCA GCA ATG AG (F) and TGA CAT CCG GTT CGT CTA CA (R). Quantitative real-time PCR was performed using SYBR Green Master Mix (TaKaRa Bio) on a TaKaRa TP-815 instrument. All expression levels were normalized to those of glyceraldehyde 3-phosphate dehydrogenase (GAPDH).

### CCL20 enzyme-linked immunosorbent assay (ELISA)

The immunoreactive CCL20 protein level in cell-culture supernatants was quantified using the enzyme-linked immunosorbent assay (ELISA) DuoSet kit (R&D Systems) according to the manufacturer’s instructions.

### Statistical analysis

All experiments were performed two or three times. Data were compared using Student's *t* test. A P-value ≤ 0.05 was considered to reflect statistical significance.

## Results

### Visfatin induced CCL20 expression and protein production in THP-1 cells

**Fig. 1:**
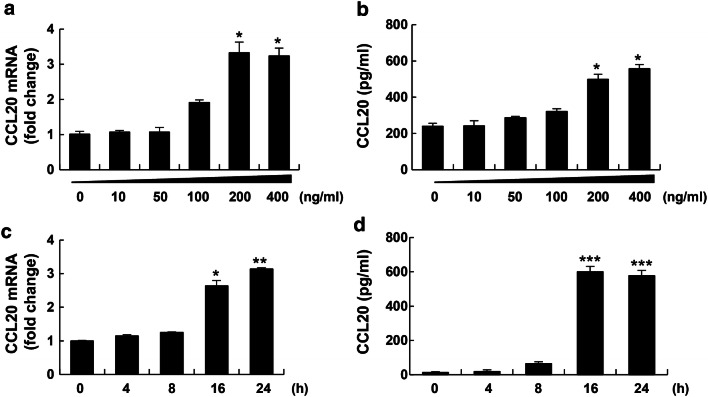
Visfatin increased CCL20 mRNA levels and secretion in THP-1 cells in a time- and dose-dependent manner. **a, b** THP-1 cells were treated for 24 h with the indicated concentrations of visfatin (0–400 ng/mL). After incubation, CCL20 mRNA levels were measured by RT-PCR (**a**) and CCL20 protein levels in cell-culture supernatants were measured by ELISA (**b**). **c, d** THP-1 cells were treated with 200 ng/mL visfatin for the indicated times (0–24 h). After incubation, CCL20 mRNA levels were measured by RT-PCR (**c**) and CCL20 protein levels in cell-culture supernatants were measured by ELISA (**d**). Data are means ± standard errors of three independent experiments. *p < 0.05, **p < 0.01, and ***p < 0.001 compared to the untreated control

CCL20 plays an important role in the pathogenesis of liver inflammation and fibrosis in NASH [9, 10]. To assess the effect of visfatin on CCL20, cells were treated with visfatin at 100 to 400 ng/mL and assayed by RT-PCR and ELISA. Visfatin at 200–400 ng/mL dramatically increased CCL20 mRNA and protein levels (Fig. 1a, b) in macrophages in a time-dependent manner (Fig. 1c, d).

### Visfatin activated NF-κB and MKK3/6-p38 signaling in THP-1 cells

**Fig. 2:**
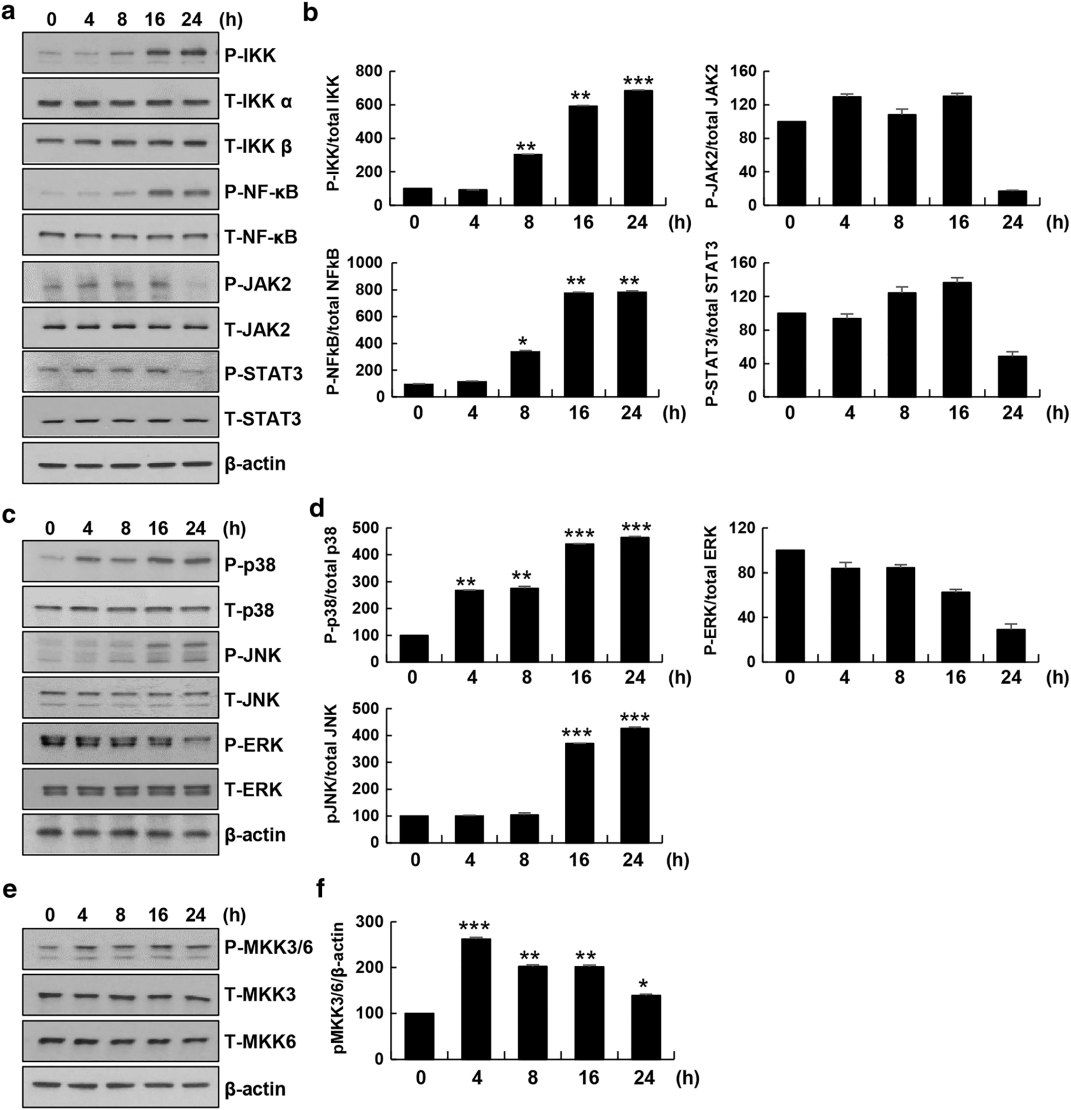
Visfatin induced activation of the NF-κB and MKK3/6-p38 MAPK signaling pathways in THP-1 cells. THP-1 cells were incubated with 200 ng/mL visfatin for the indicated times**. a, b** IKK/NF-κB signaling was analyzed using anti-phospho-IKKα/β and -phospho-NF-κB antibodies. JAK/STAT3 signaling was analyzed using anti-phospho-JAK2, -phospho-STAT3, and β-actin antibodies. **c, d** MAP kinase signaling was analyzed using anti-phospho-p38, -phospho-JNK, -phospho-ERK, and β-actin antibodies. **e, f** The MAPK signaling pathway consisting of MKK3/6 was analyzed using anti-phospho-MKK3/6 and β-actin antibodies. *p < 0.05, **p < 0.01, and ***p < 0.001 compared to the untreated control. The control phosphoprotein intensity was set to 100%, and relative test intensities were calculated. Data are means ± standard errors of three independent experiments

It has been reported that CCL20 expression is regulated by signaling pathways such as the NF-κB, STAT3, and stress-mediated MAPK signaling pathways under various conditions [22–24]. To explore whether visfatin affected IKK/NF-κB, JAK/STAT, and stress-mediated MAPK signaling, macrophages were treated with visfatin for the indicated times. Next, we evaluated the effect of visfatin in macrophages by immunoblotting. Visfatin stimulated IKK/NF-κB activation in a time-dependent manner but did not impact JAK/STAT activation (Fig. 2a, b). Next, we examined whether visfatin activated the MAPK p38, JNK, and ERK pathways. Activation of p38 in a time-dependent manner was detected. Visfatin increased JNK pathway activation at later time points but did not affect activation of the ERK pathway (Fig. 2c, d). Activation of MKK3 and MKK6, upstream kinases of p38, was increased by visfatin (Fig. 2e, f). Thus, visfatin induced activation of the MKK3/6-p38 and NF-κB signaling pathways in THP-1 cells.

### NF-κB and MLK3-p38 MAPK inhibition attenuated visfatin-induced expression of CCL20

**Fig. 3:**
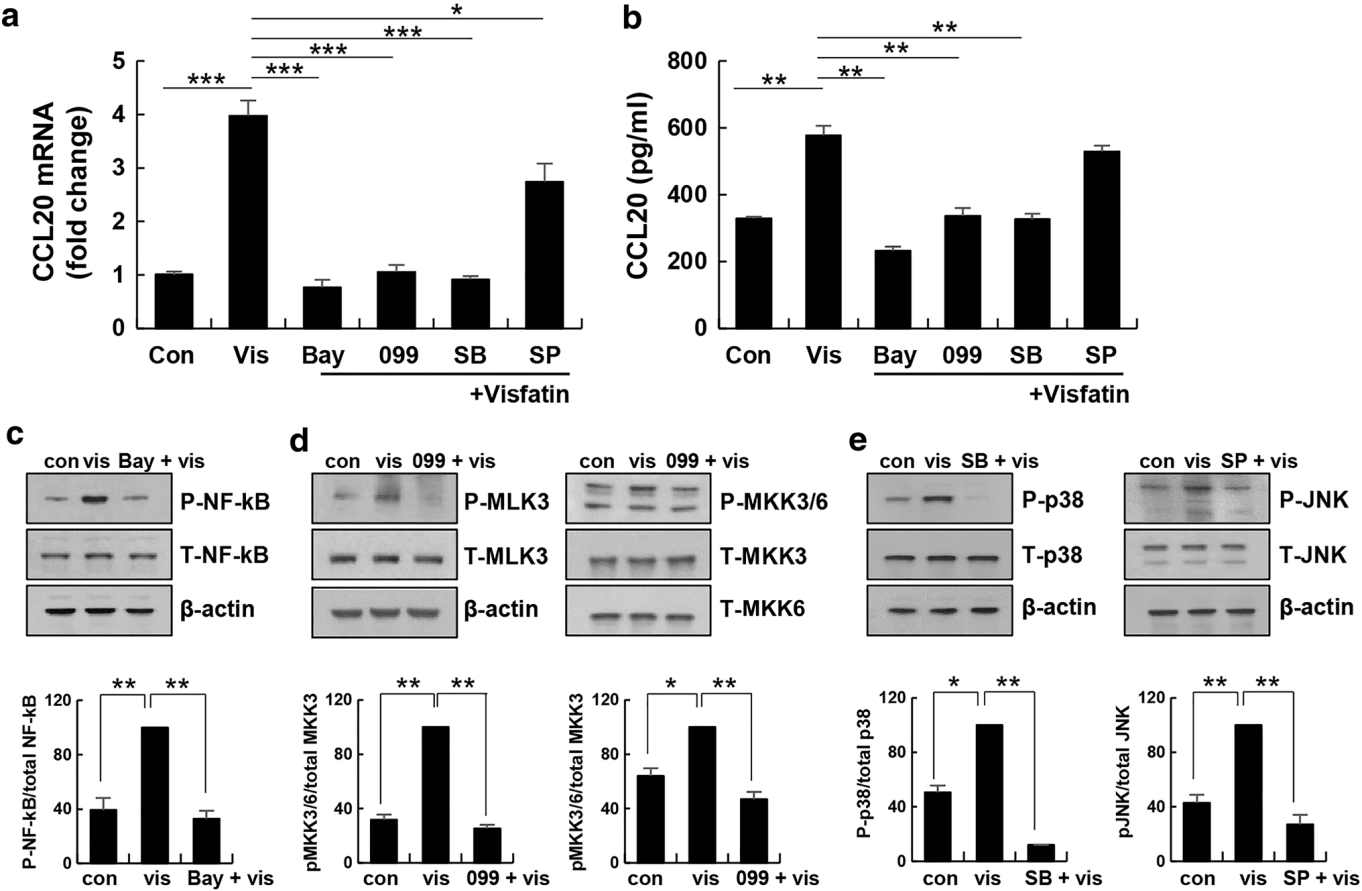
Effects of signaling inhibitors on visfatin-induced CCL20 expression and release. THP-1 cells were pre-incubated with 5 µM BAY11-7082 (NF-κB inhibitor), 1 µM URMC-099 (MLK3 inhibitor), 10 µM SB 203,580 (p38 MAPK inhibitor), and 10 µM SP 600,125 (JNK inhibitor) for 4 h, and stimulated with visfatin (vis) for 20 h. **a** Expression of CCL20 was analyzed by real-time PCR. **b** CCL20 level in culture supernatants were measured by ELISA. Data are means ± standard errors of three independent experiments. **c–e** NF-κB, MLK3, MKK3/6, p38, and JNK signaling was measured using antibodies against phospho-NF-κB, phospho-MLK3, phospho-MKK3/6, phospho-p38, phospho-JNK, and β-actin. The maximum phosphoprotein intensity in visfatin-treated samples was set to 100%, and the relative intensities of test samples were calculated. The maximum control phosphoprotein intensity was set to 100%, and relative test intensities were calculated. Data are means ± standard errors of three independent experiments. *p < 0.05, **p < 0.01, and ***p < 0.001 compared to the control or visfatin

Because visfatin stimulated NF-κB and MKK3/6-p38 MAPK signaling, we investigated whether the expression of CCL20 induced by visfatin is associated with these signaling pathways in THP-1 cells. THP-1 cells were pretreated with an NF-κB, MLK3, p38, or JNK inhibitor followed by the addition of visfatin. Expression of CCL20 was assessed by RT-PCR and ELISA. BAY11-7082, URMC-099, and SB 203,580 blocked visfatin-induced CCL20 expression (Fig. 3a) and secretion (Fig. 3b). SP 600,125 did not block CCL20 protein secretion. To confirm the degree of inhibition, stimulated cells were assessed by immunoblotting. Visfatin increased NF-κB phosphorylation approximately two-fold, but BAY11-7082 completely blocked it (Fig. 3c). Visfatin increased MLK3 and MKK3/6 phosphorylation; this phosphorylation was blocked by URMC-099 (Fig. 3d). Visfatin increased p38 and JNK phosphorylation, which was blocked by SB 203,580 and SP 600,125 (Fig. 3e). Therefore, visfatin induced CCL20 expression by activating MKK3/6-p38 and NF-κB, but not JNK, signaling in THP-1 cells.

### Visfatin-induced CCL20 expression increased fibrosis markers in HSCs

**Fig. 4:**
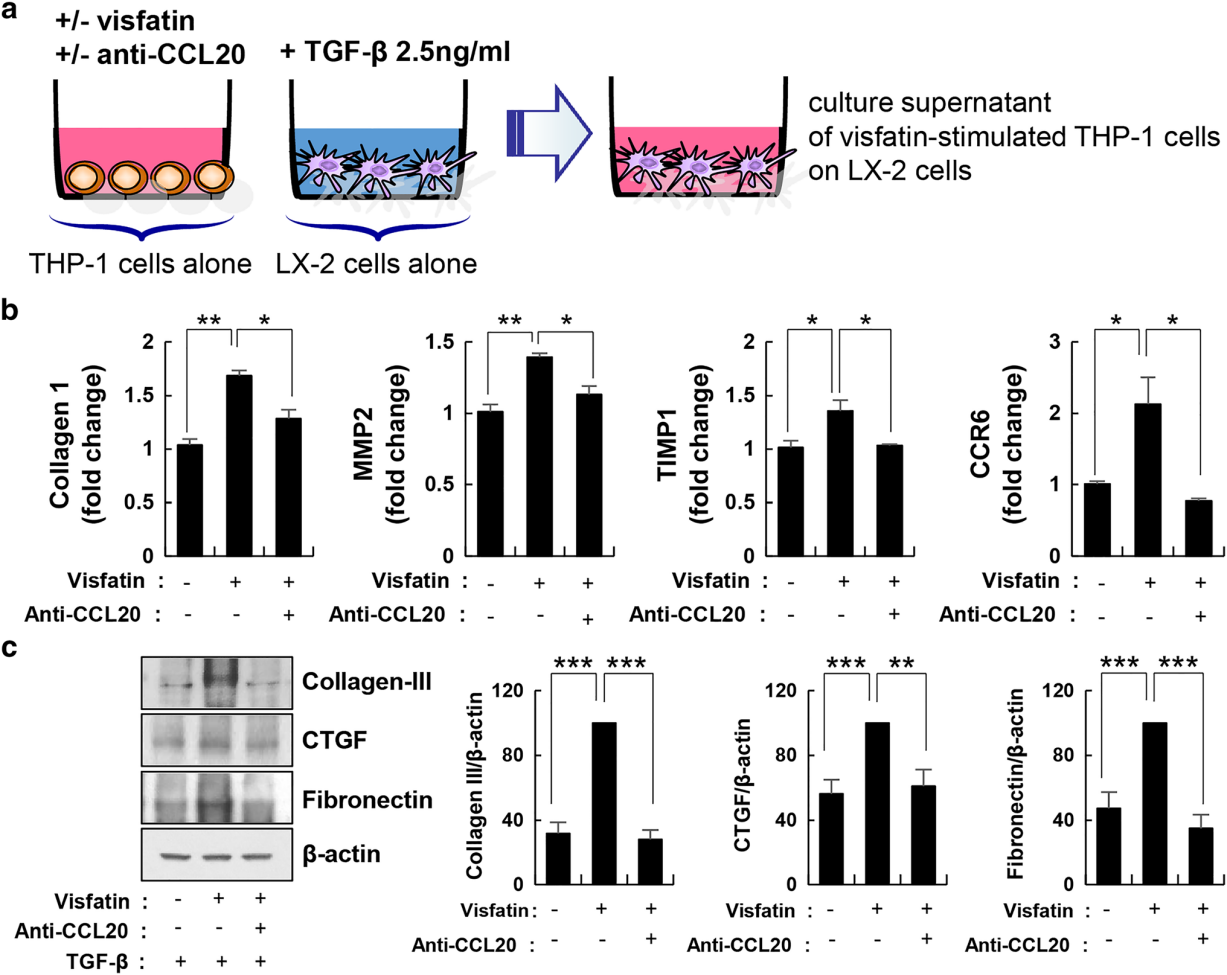
Fibrosis markers were increased by visfatin-induced CCL20 expression in hepatic stellate cells. **a** THP-1 cells were stimulated by visfatin for 24 h and exposed or not to an anti-CCL20 antibody for 1 h before harvest. Human LX-2 hepatic stellate cells were stimulated with TGF-β (2.5 ng/mL) for 24 h. Next, the culture medium of LX-2 cells was removed and the culture supernatant of THP-1 cells was added for 24 h. **b** Collagen I, MMP2, TIMP1, and CCR6 mRNA levels measured by real-time PCR. Data are means ± standard errors of three independent experiments. **c** Fibronectin, CTGF, and collagen III were assayed by immunoblotting using specific antibodies. The maximum control phosphoprotein intensity was set to 100%, and relative test intensities were calculated. Data are means ± standard errors of three independent experiments. *p < 0.05, **p < 0.01, and ***p < 0.001 compared to the control

Because HSCs are key players in the development of liver fibrosis, we investigated the biological effect of CCL20, which is secreted from visfatin-treated macrophages, on LX-2 cells. LX-2 cells were stimulated with the culture supernatant of macrophages stimulated with visfatin. Expression of fibrosis markers was assessed by RT-PCR and immunoblotting. The levels of the fibrosis markers collagen I, matrix metalloproteinase (MMP) 2, and tissue inhibitor of matrix metalloproteinase (TIMP) were increased in LX-2 cells, as was that of CCR6, a CCL20 receptor. To assess the role of CCL20 in fibrosis, we blocked CCL20 using a neutralizing antibody (Fig. 4a). The anti-CCL20 neutralizing antibody significantly decreased the mRNA levels of collagen I, MMP2, TIMP1, and CCR6 (Fig. 4b). In addition, activation of collagen III, CTGF, and fibronectin was significantly reduced (Fig. 4c). Therefore, CCL20 secreted by macrophages might be a key mediator of fibrosis.

## Discussion

In the present study, we found that visfatin significantly increased CCL20 expression and protein secretion in THP-1 cells via the NF-κB and MKK3/6-p38 pathways. Also, visfatin-induced CCL20 expression increased the levels of the fibrosis markers including collagen I and III, MMP2, TIMP1, CTGF, and fibronectin, which were reduced by neutralization of CCL20, in HSCs.

Progression from NAFLD to NASH is no longer considered a simple hepatic disease and involves complex immune mechanisms [1]. Immune cells such as lymphocytes, Kupffer cells, and dendritic cells secrete cytokines and chemokines to promote fibrosis [2]. Macrophages are abundant in the liver and are critical for the development of NAFLD [25]. Hepatic macrophages, such as Kupffer cells and monocyte-derived macrophages, accumulate mainly at sites of inflammation, and secrete cytokines and chemotactic agents or act directly as effectors [25]. Animal models of liver fibrosis have provided evidence for an interaction between inflammatory signaling pathways, monocytes/macrophages, and HSCs in driving fibrogenesis [26]. HSC activation is a major cause of liver fibrosis and activated HSCs produce type I and III collagen and fibronectin in the extracellular matrix [27]. Macrophage/HSC crosstalk plays an important role in the progression of NAFLD to NASH. Here, we focused on the mediation by CCL20 of the interaction between macrophages and HSCs.

The release of CCL20 is a key feature of chronic inflammatory diseases [4]. Under inflammatory conditions, epithelial cells, endothelial cells, fibroblasts, and monocytes are important sources of CCL20 [28–30]. Chu et al. [9] reported that expression of CCL20, an important inflammatory mediator, is increased in NAFLD fibrosis. Affo et al. [10] reported that CCL20 exerted proinflammatory and profibrogenic effects in cultured primary HSCs. Therefore, CCL20 induced by palmitic acid or LPS is involved in liver fibrosis [9, 10]. Here, we showed that visfatin, an adipocytokine, induced CCL20 expression in macrophages and activated HSCs.

Visfatin modulates inflammatory cytokines in the association with obesity, insulin resistance, and cardiovascular disease, and plays an important role in the pathogenesis of inflammation-related diseases [31]. We previously evaluated the effects of visfatin on inflammation and insulin resistance in HepG2 cells and the molecular mechanisms involved [32]. In addition, several studies have reported an association between the circulating visfatin level and NAFLD [33, 34], and some studies have described increases in liver visfatin expression levels in NAFLD [35, 36]. In our results, visfatin induced CCL20 expression and secretion in macrophages. Also, CCL20 in co-culture increased the expression of the fibrotic markers, collagen I and III, MMP2, TIMP1, CTGF, and fibronectin in HSCs, and neutralization of CCL20 led to decreased fibrosis marker expression in HSCs. CCL20 is the only CCR6-binding chemokine and cannot react with other known chemokine receptors [8]. CCL20–CCR6 interactions are involved in several inflammatory processes, such as rheumatoid arthritis, multiple sclerosis, and liver fibrosis [8, 9, 37]. Stimulation by CCL20 upregulated the mRNA level of CCR6 in hepatocellular carcinoma cells [38]. Interestingly, in our study, the increased expression of CCR6 in HSCs treated with the supernatant of visfatin stimulated in macrophages were decreased by neutralization of CCL20.

Next, we confirmed the signaling pathway involved in the upregulation of CCL20 by visfatin in macrophages. Visfatin enhances CXCL8, CXCL10, and CCL20 production via NF-κB in human keratinocytes [39]. NF-κB and STAT3 are important transcription factors in inflammatory signaling, and we reported that visfatin causes inflammation by activating STAT3 and NF-κB in hepatocytes [32]. Visfatin induced production of CCL20 via NF-κB, but not STAT3, in macrophages. The production of CCL20 has been reported to activate MAPK and PI3K signaling pathways following IL-1β treatment of lung cancer cells [40]. We examined whether visfatin regulated CCL20 via MAPK signaling pathways in macrophages. The MAPK family is characterized by ERK, JNK, and p38 kinases. MAPKs are linked to a protein kinase cascade. Stimulation of a mitogen-activated protein kinase kinase kinase (MAP3Ks, MAPKKKs) leads to the phosphorylation and activation of MAP kinase kinases (MAP2Ks, MKKs), which stimulate MAPK activity. The phosphorylation of JNK is mediated by MKK4/7, and that of p38 by MKK3/6 [41]. Following treatment with visfatin, the activity of ERK was unchanged, the activity of JNK was increased at later time points, and the activity of p38 was increased at earlier time points. So, we investigated the activity of MKK3 and MKK6, the upstream kinases of p38. Visfatin induced CCL20 expression via the MKK3 or MKK6 signaling pathway in macrophages. The STAT3, JNK, and ERK pathways were not involved in visfatin-induced CCL20 expression in macrophages in a manner involving the NF-κB and MKK3/6-p38 pathways. The involvement of different pathways in the induction of CCL20 expression may depend largely on the cell type and stimulus.

In summary, visfatin, an important mediator of inflammation, increased the expression of CCL20 via the NF-κB and MKK3/6-p38 signaling pathways in macrophages. In addition, visfatin-induced CCL20 expression in macrophages promoted the expression of fibrosis markers in HSCs. These data suggest induction of CCL20 expression by visfatin in macrophages might play an important role in the development of hepatic fibrosis. Further in vivo studies are required to explore how visfatin affects NAFLD progression, considering that NAFLD is a multifactorial liver disease with a very complex pathogenesis.
